# COVID-19 Bereavement in Ten Latin American Countries: Measurement Invariance of the Pandemic Grief Scale and Its Relation to Suicidal Ideation

**DOI:** 10.1177/00302228211048566

**Published:** 2021-10-19

**Authors:** Tomás Caycho-Rodríguez, Pablo D. Valencia, Lindsey W. Vilca, Sherman A. Lee, Carlos Carbajal-León, Andrea Vivanco-Vidal, Daniela Saroli-Araníbar, Mario Reyes-Bossio, Michel White, Claudio Rojas-Jara, Roberto Polanco-Carrasco, Miguel Gallegos, Mauricio Cervigni, Pablo Martino, Diego Alejandro Palacios, Rodrigo Moreta-Herrera, Antonio Samaniego-Pinho, Marlon Elías Lobos Rivera, Andrés Buschiazzo Figares, Diana Ximena Puerta-Cortés, Ibraín Enrique Corrales-Reyes, Raymundo Calderón, Bismarck Pinto Tapia, Ilka Franco Ferrari, Carmen Flores-Mendoza, Walter L. Arias Gallegos

**Affiliations:** 1 Facultad de Ciencias de la Salud, 33220 Universidad Privada del Norte, Universidad Privada del Norte, Lima, Perú; 2 Facultad de Estudios Superiores Iztacala, 7180 Universidad Nacional Autónoma de México, Universidad Nacional Autónoma de México, Tlanepantla de Baz, State of Mexico, Mexico; 3 Departamento de Psicología, Universidad Peruana Unión, Lima, Perú; 4 Department of Psychology, 6013 Christopher Newport University, Christopher Newport University, Newport News, Virginia, United States; 5 Facultad de Psicología, 33217 Universidad Peruana de Ciencias Aplicadas, 33217 Universidad Peruana de Ciencias Aplicadas, Lima, Perú; 6 Dirección General de Investigación, 33218 Universidad Peruana Unión, Universidad Peruana Unión, Lima, Perú; 7 Facultad de Ciencias de la Salud, Departamento de Psicología, Universidad Católica del Maule, Talca, Chile; 8 Cuadernos de Neuropsicología, Centro de Estudios Académicos en Neuropsicología, Rancagua Chile; 9 Pontificia Universidade Católica de Minas Gerais, Minas Gerais, Brasil; 10 Consejo Nacional de Investigaciones Científicas y Técnicas, Rosario, Argentina; 11 Centro Interdisciplinario de Investigaciones en Ciencias de la Salud y del Comportamiento, 28179 Universidad Adventista del Plata, Universidad Adventista del Plata, Consejo Nacional de Investigaciones Científicas y Técnicas, Rosario, Argentina; 12 Centro de Investigación en Neurociencias de Rosario, Facultad de Psicología, 28237 Universidad Nacional de Rosario, 28237 Universidad Nacional de Rosario, Rosario, Argentina; 13 Centro de Desarrollo Humano, Universidad Mariano Gálvez, Guatemala, Guatemala; 14 Escuela de Psicología, 27884 Pontificia Universidad Católica del Ecuador, Pontificia Universidad Católica del Ecuador, Ambato, Ecuador; 15 Carrera de Psicología, Facultad de Filosofía, 187173 Universidad Nacional de Asunción, Universidad Nacional de Asunción, Asunción, Paraguay; 16 Escuela de Psicología, Facultad de Ciencias Sociales, 34872 Universidad Tecnológica de El Salvador, Universidad Tecnológica de El Salvador, San Salvador, El Salvador; 17 Instituto Alfred Adler Uruguay, Centro de Estudios Adlerianos, Montevideo, Uruguay; 18 Programa de Psicología, 27950 Universidad de Ibagué, Universidad de Ibagué, Ibagué, Colombia; 19 Servicio de Cirugía Maxilofacial, Hospital General Universitario Carlos Manuel de Céspedes, Universidad de Ciencias Médicas de Granma, Bayamo, Granma, Cuba; 20 Carrera de Psicología, Facultad de Ciencias de la Salud, 27822 Universidad del Valle de México, Universidad del Valle de México, Ciudad de México, México; 21 Carrera de Psicología, Universidad Católica Boliviana San Pablo, La Paz, Bolivia; 22 Facultad de Filosofía y Ciencias Humanas. Universidad Federal de Minas Gerais, Minas Gerais, Brasil; 23 Departamento de Psicología, Universidad Católica San Pablo, Arequipa, Perú

**Keywords:** invariance, cross-cultural, grief, COVID-19, Latin America

## Abstract

The present study aimed to evaluate the cross-cultural measurement invariance of the Pandemic Grief Scale (PGS) in ten Latin American countries. A total of 2,321 people who had lost a family member or other loved one due to COVID-19 participated, with a mean age of 34.22 years old (SD = 11.99). In addition to the PGS, a single item of suicidal ideation was applied. The unidimensional model of the PGS had adequate fit in most countries and good reliability estimates. There was evidence of measurement invariance by country and gender. Also, a one-point increase in the PGS was associated with an almost twofold increase in the odds of suicidal ideation. Scores greater than or equal to 4 on the PGS are proposed as a cut off to identify individuals with suicidal ideation. Strong evidence of the cross-cultural validity of the PGS is provided.

## Introduction

The COVID-19 pandemic is one of the largest health crises in contemporary history (Albuquerque et al., 2021), generating more than 171 million diagnosed cases and more than 3.5 million deaths. In the case of Latin America, as of June 29, 2021, there are a total of 37,208,956 registered cases of COVID-19, with Brazil being the country most affected by this pandemic in the region, with about 18.4 million confirmed cases, followed by Argentina with approximately 4.4 million infected and Mexico with a total of 2,507,453 cases. Colombia, Peru, Chile and Ecuador are also among the countries most affected by the new type of coronavirus in Latin America. Likewise, as of the same date, almost 1.3 million people had died due to the COVID-19 pandemic in Latin America, where the majority of fatal cases occurred in Brazil, with a total of 514,092 deaths, followed by Mexico, with 232,608 deaths. This situation has led to efforts being focused mainly on overcoming the virus (Scholten et al., 2020); however, not much attention has been given to the grief of people who have lost a loved one to COVID-19. It is now suggested that there is an urgent need to prepare for a possible “grief pandemic” due to COVID-19 (Weinstock et al., 2021).

Grief is a painful but expected emotional response to loss that is present in all human cultures (Weinstock et al., 2021). Grief due to the death of loved ones during the COVID-19 pandemic is estimated to be more severe compared to pre-pandemic grief (Eisma & Tamminga, 2021). In this regard, prior to the COVID-19 pandemic, about 10% of people who have lost a loved one had complications in the grieving process (Lundorff et al., 2017). Currently, the COVID-19 pandemic is generating an increase in the prevalence of dysfunctional grief worldwide (Eisma et al., 2020). It is estimated that the death of one person from COVID-19 would emotionally affect about 9 family members (Verdery et al., 2020) and that in total there are approximately 16 million bereaved people worldwide (Tang, & Xiang, 2021). This increase in the prevalence of dysfunctional grief is associated with the characteristics that accompany death from COVID-19, such as “grief overload” due to the increase in deaths of family members and other loved ones, which interferes with the ability to cope, as well as restrictions that prohibit visiting relatives or loved ones in hospitals or intensive care units, accompanying them in their last days of life and performing funeral ceremonies, which can generate or increase feelings of guilt (Kokou-Kpolou et al., 2020). However, it is also important to note that these pandemic-related difficulties may also have produced feelings of solidarity among some survivors who were comforted by their feelings of unity and shared adversity (Juth et al., 2015). Studies have shown that bereaved people who have greater contact with family and friends, whether the support is through technology or in person, exhibit better quality of life and well-being (Burke & Neimeyer, 2012).

Bereaved persons tend to experience feelings of denial, anger and guilt, bargaining, depression, and acceptance at the onset of loss (Maciejewski et al., 2007). However, when grief symptoms are prolonged, obstructed, intensified or delayed (unresolved grief), it can become a psychiatric problem. (Wallace et al., 2020), due to the inability to say goodbye to the deceased, excessive levels of guilt, and lack of social support (Mortazavi et al., 2020). This can negatively impact family, work, and general social relationships for several months after the death of the loved one (Menzies et al., 2020). Likewise, the grief experience can generate physical health risks, such as the onset of cardiac and immune system problems, addictions, deterioration of overall quality of life and even suicide (Maercker et al., 2017). Regarding this last point, there is evidence that dysfunctional grief associated with the death of a loved one during the COVID-19 pandemic leads to increased suicidal ideation and attempts (Halford et al., 2020; Reger et al., 2020; Wand et al., 2020). Moreover, suicide has long been recognized as a serious global public health concern, as it is responsible for more deaths worldwide than malaria, breast cancer, war, and homicide (World Health Organization, 2019).

This indicates that the prevalence of grief related to COVID-19 and its consequences for physical and mental health are an important public health problem that countries around the world must face (Doka, 2021; Verdery & Smith-Greenway, 2020); therefore, grief care should be considered an important part of health and social care (Pearce et al., 2021). However, the absence of a valid instrument to assess grief due to the loss of a loved one produced by the COVID-19 pandemic means that its symptoms may not be recognized or are misdiagnosed (for example, as symptoms of depression) and, therefore, are not treated or are treated with general and ineffective interventions (van Eersel et al., 2019). Seeking to fill this instrumental gap, Lee and Neimeyer (2020), developed the Pandemic Grief Scale (PGS) to quickly identify possible cases of dysfunctional grief due to COVID-19-related loss. The PGS is comprised of five items and was validated in a sample of 831 U.S. adults who experienced the death of a person due to COVID-19. The original validation study concluded that the PGS measures the COVID-19 grief construct in a unidimensional model and is reliable (α = .86) and invariant across different gender, age and race groups; it also exhibits adequate diagnostic properties (87% sensitivity and 71% specificity), comparable or superior to other psychiatric measures (Lee & Neimeyer, 2020).

Likewise, the PGS has been translated and validated in Turkish (Evren et al., 2021), Polish (Skalski et al., 2021) and Spanish (Caycho-Rodríguez et al., 2021a), confirming in all contexts its adequate psychometric properties. However, no evidence has been provided on the equivalence of PGS measurement between different countries, which is crucial to test the cross-cultural applicability of theoretical models of grief related to COVID-19 (Sue, 1999). In this regard, measurement invariance (MI) would indicate whether the construct dysfunctional COVID-19 pandemic grief retains its meaning across different groups, which is important when comparing groups using psychological measures (Millsap & Kwok, 2004). Making comparisons between groups with non-invariant measures would lead to biased results and inappropriate interpretations of mean differences between groups (Hoyle & Smith, 1994). Thus, differences in instrument scores would not reflect true differences in the construct being measured (Cheung & Rensvold, 2002).

A study to establish MI for a COVID-19-related measure of grief is important considering that it is a complex process, shaped by individual experiences, such as the person’s understanding of the meaning of death and the relationships between the living and the dead (Neimeyer et al., 2014), which can be shaped by cultural factors (Silverman et al., 2021). This is corroborated by cross-cultural studies indicating differences in the way people grieve (Smid et al., 2018). Thus, bereavement is an individual experience that would also be influenced by the social norms of each culture (Lund, 2021). In this sense, the way in which people handle the death of a loved one and the emotional experience that this entails also depends on the cultural context in which it occurs (Jakoby, 2012). For example, experiencing pain and grief in a context such as Latin America, characterized by stressors such as inequality, poverty and high prevalence of chronic diseases (Pablos-Méndez et al., 2020), as well as a high number of people diagnosed with and killed by COVID-19 (e Silva, & Pitzurra, 2020; Muñoz, 2020) can negatively affect people's ability to accept the loss of loved ones (Kim et al., 2017). However, despite these cultural differences, there may be enough similarities in some of the symptoms of dysfunctional grief that people from different cultures may find that a screening tool such as the PGS (Lee & Neimeyer, 2020) would be of universal application.

Accordingly, the study of cross-cultural MI would also allow for a comprehensive assessment of mental health associated with grief. Smid et al. (2018) indicate that a cultural perspective of grief assessment for the loss of loved ones would allow health care professionals to: (1) understand cultural norms linking grief to the onset of mental disorders; (2) facilitate exploration of the psychological consequences of not having been able to perform rituals to say goodbye to loved ones and the role of cultural traditions and beliefs about death in maintaining or increasing distress; (3) learn expectations about the most appropriate type of help; (4) facilitate decision making regarding intervention practices and the integration of culturally appropriate rituals.

Taking the above into consideration, and as part of a broader project evaluating the psychometric properties of different instruments measuring mental health indicators during the COVID-19 pandemic (see, for example, Caycho-Rodríguez et al., 2021b), the present study aimed to evaluate the cross-cultural MI of the PGS in ten Latin American countries, namely Brazil, Bolivia, Chile, Colombia, Ecuador, El Salvador, Guatemala, Mexico, Paraguay, and Peru. Evaluating the MI of the PGS would provide evidence to support the generalizability of the measure to different cultures besides that in which it was developed, supporting in turn the universality of the construct across different cultural groups. Given previous evidence indicating the presence of a unifactorial structure (Evren et al., 2021; Lee & Neimeyer, 2020; Skalski et al., 2021), the presence of at least partial invariance would be expected. Additionally, the prevalence of dysfunctional grief related to COVID-19 was assessed as well as the ability of the PGS to predict suicidal ideation cross-sectionally. Regarding prevalence, studies indicate that between 56.6% and 66% of US persons present clinically relevant COVID-19-related grief (Lee & Neimeyer, 2020; Lee et al., 2021). Therefore, with Latin America being one of the regions most affected by COVID-19, similar prevalence percentages would be expected. Finally, as indicated by the literature on suicide (Halford et al., 2020; Hill et al., 2021; Reger et al., 2020; Wand et al., 2020), grief from the death of a loved one during the COVID-19 pandemic is expected to be associated with an increased likelihood of suicidal ideation. The link between dysfunctional grief and suicidal ideation during the COVID-19 pandemic is particularly important to examine given that suicide continues to be one of the leading causes of death worldwide (World Health Organization, 2019). Moreover, assessing this association in different countries is valuable if we consider that public spending, financial resources available to citizens, medical care, and variations in social support may influence the likelihood of suicidal ideation (Cheung et al., 2021).

## Method

### Participants

Participants were 2,321 people who had lost a family member or other loved one due to COVID-19 from 9 Spanish-speaking Latin American countries (Bolivia, Brazil, Chile, Colombia, Ecuador, El Salvador, Guatemala, Mexico, Paraguay, Peru) and 1 Portuguese-speaking country (Brazil), selected non-probabilistically by convenience. All participants had to be of legal age, have suffered the death of a family member or friend from COVID-19, and give informed consent to participate in the study. The minimum number of participants in each country was calculated with Soper software (Soper, 2021), taking into account the number of observed variables (5 items), latent variables of the model to be evaluated (dysfunctional grief related to COVID-19), the anticipated effect size (λ = 0.3), the probability (α = 0.05) and the statistical power (1 − β = 0.95).

The average number of participants in each country was 232 people and ranged from 156 (Bolivia) to 441 (Paraguay). A total of 708 women, 1,721 men, and 7 transgender people participated, with an average age of 34.22 years old (SD = 11.99), where the Ecuadorian sample was the youngest (M = 28.81, SD = 10.65) and the sample from Guatemala had the highest average age (M = 41.57, SD = 12.90). Furthermore, 448 participants indicated having been diagnosed with COVID-19 (19.30%) and 389 (16.76%) reported having had suicidal ideation. More detailed demographic information for each country is shown in Table 1.

**Table 1.** Demographic Information of the Participants in Each Country.

	Bolivia (*n* = 156)	Brazil (*n* = 206)	Chile (*n* = 179)	Colombia (*n* = 215)	Ecuador (*n* = 295)	El Salvador(*n* = 437)	Guatemala (*n* = 171)	Mexico (*n* = 202)	Paraguay (*n* = 441)	Peru (*n* = 234)
Gender (%)										
Female	56 (35.9)	49 (23.8)	45 (25.1)	69 (32.1)	98 (33.2)	167 (38.2)	65 (38.0)	80 (39.6)	111 (25.2)	68 (29.1)
Male	99 (63.5)	157 (76.2)	134 (74.9)	146 (67.9)	197 (66.8)	268 (61.3)	106 (62.0)	120 (59.4)	328 (74.4)	166 (70.9)
Transgender/ Nonbinary	1 (0.6)	0 (0.0)	0 (0.0)	0 (0.0)	0 (0.0)	2 (0.5)	0 (0.0)	2 (1.0)	2 (0.4)	0 (0.0)
Age (M ± SD)	40.60 ± 14.74	35.23 ± 10.83	38.45 ± 12.61	30.65 ± 13.04	28.81 ± 10.65	29.49 ± 9.05	41.57 ± 12.90	33.89 ± 14.22	31.59 ± 11.02	31.97 ± 10.87
Marital status (%)										
Single	71 (45.5)	108 (52.4)	80 (44.7)	154 (71.6)	205 (69.5)	315 (72.1)	70 (40.9)	113 (55.9)	285 (64.6)	147 (62.8)
Married	52 (33.3)	57 (27.7)	53 (29.6)	35 (16.3)	61 (20.7)	79 (18.1)	69 (40.4)	70 (34.6)	106 (24.0)	51 (21.8)
Divorced	21 (13.5)	16 (7.8)	16 (8.9)	6 (2.8)	18 (6.1)	9 (2.1)	15 (8.8)	12 (5.9)	14 (3.2)	8 (3.4)
Cohabiting	7 (4.49)	23 (11.2)	27 (15.1)	17 (7.9)	7 (2.4)	33 (7.6)	12 (7.0)	4 (2.0)	33 (7.5)	26 (11.1)
Widowed	5 (3.21)	2 (1.0)	3 (1.7)	3 (1.4)	4 (1.4)	1 (0.2)	5 (2.9)	3 (1.5)	3 (0.7)	2 (0.9)
Higher education (%)										
No	7 (4.5)	30 (14.6)	12 (6.7)	46 (21.4)	53 (18.0)	118 (27.0)	16 (9.4)	14 (6.9)	51 (11.6)	15 (6.4)
Yes, technical levelª	5 (3.2)	70 (34.0)	21 (11.7)	24 (11.2)	8 (2.7)	26 (6.0)	14 (8.2)	34 (16.8)	16 (3.6)	17 (7.3)
Yes, university levelª	144 (92.3)	106 (51.5)	146 (81.6)	145 (67.4)	234 (79.3)	293 (67.0)	141 (82.5)	154 (76.2)	374 (84.8)	202 (86.3)
Job type (%)										
Permanent	67 (43.0)	135 (65.5)	107 (59.8)	71 (33.0)	108 (36.6)	221 (50.6)	120 (70.2)	94 (46.5)	252 (57.1)	92 (39.3)
Temporary	35 (22.4)	15 (7.3)	23 (12.8)	36 (16.7)	46 (15.6)	65 (14.9)	28 (16.4)	37 (18.3)	69 (15.6)	50 (21.4)
Unemployed	54 (34.6)	56 (27.2)	49 (27.4)	108 (50.2)	141 (47.8)	151 (34.6)	23 (13.4)	71 (35.2)	120 (27.2)	92 (39.3)
Had COVID-19 (%)										
Yes	48 (30.8)	41 (19.9)	14 (7.8)	42 (19.5)	53 (18.0)	72 (16.5)	14 (8.2)	38 (18.8)	69 (15.6)	57 (24.4)
No	82 (52.6)	107 (51.9)	151 (84.4)	120 (55.8)	187 (63.4)	211 (48.3)	136 (79.5)	125 (61.9)	283 (64.2)	124 (53.0)
Maybe yes	17 (10.9)	26 (12.6)	7 (3.9)	34 (15.8)	33 (11.2)	119 (27.2)	12 (7.0)	17 (8.4)	50 (11.3)	37 (15.8)
Maybe no	9 (5.8)	32 (15.5)	7 (3.9)	19 (8.8)	22 (7.5)	35 (8.0)	9 (5.3)	22 (10.9)	39 (8.8)	16 (6.84)
Suicidal ideation										
No	136 (87.2)	183 (88.8)	160 (89.4)	186 (86.5)	230 (78.0)	355 (81.2)	147 (86.0)	172 (85.2)	381 (86.4)	197 (84.2)
Yes	20 (12.8)	23 (11.2)	19 (10.6)	29 (13.5)	65 (22.0)	82 (18.8)	24 (14.0)	30 (14.8)	60 (13.6)	37 (15.8)

^a^Both complete and incomplete studies were included.

### Instruments

#### Sociodemographic Data

The survey provided information on age, sex, marital status, educational level, employment and COVID-19 diagnosis.

#### Pandemic Grief Related to COVID-19

The Pandemic Grief Scale (PGS; Lee & Neimeyer, 2020) assesses symptoms of dysfunctional grief related to the death of a loved one due to COVID-19 in the past two weeks. The PGS is comprised of 5 items that have four response options, (0 = “not at all” to 3 = “almost every day”). The total score of the PGS ranges from 0 to 25, where higher values indicate a higher frequency of dysfunctional grief symptoms. Similarly, a score equal to or greater than 7 allows for adequate discrimination between people with and without dysfunctional grief related to COVID-19. The PGS was translated into Brazilian Portuguese using the back-translated method. Initially, two independent researchers, one content expert, familiar with the COVID-19 and bilingual in English and Portuguese, and another native English language expert, translated the PGS from English to Portuguese. Then, two different researchers, one a content expert and the other a language expert, who were not familiar with the first translation, translated the Portuguese version back into English. Both versions were compared and evaluated for possible inconsistencies to produce a harmonized version. This version was subjected to a pilot test with the participation of 15 Portuguese-speaking people, and any inconsistencies in phrasing were rectified in order to have a final version in Brazilian Portuguese. The same procedure was followed for the translation of the PGS from English to Spanish, as published in Caycho-Rodríguez et al. (2021a). See Table 2 for the final Spanish and Portuguese version of the PGS.

**Table 2.** *Portuguese and Spanish Version of the Pandemic Grief Scale*.

Portuguese version	Spanish version
Nas últimas 2 semanas, quantas vezes você experimentou os seguintes pensamentos, sentimentos, comportamentos relacionados à sua perda?	Durante las últimas 2 semanas, ¿con qué frecuencia ha experimentado los siguientes pensamientos, sentimientos o comportamientos relacionados con su pérdida?
Ítem 1: Eu queria morrer para estar com o falecido	Ítem 1: Quería morir para estar con la persona que falleció
Ítem 2: Experimentei confusão sobre meu papel na vida ou senti que minha identidade foi diminuída por causa da perda	Ítem 2: Experimenté confusión sobre mi papel en la vida o sentí que mi identidad había cambiado debido a la pérdida
Ítem 3: Nada parecia importar muito para mim por causa dessa perda.	Ítem 3: Nada parecía importarme debido a esta pérdida
Ítem 4: Achei difícil ter lembranças positivas sobre o falecido	Ítem 4: Me resultó difícil tener recuerdos positivos de la persona fallecida
Ítem 5: Eu acreditava que sem o falecido a vida seria sem sentido, vazia ou não poderia continuar	Ítem 5: Creía que, sin la persona fallecida, la vida carecía de sentido, estaba vacía o no podía continuar

#### Suicidal Ideation

A single item used in the Lee and Neimeyer (2020) study was used to assess suicidal ideation during the past 2 weeks. The content of the single item is “I wish I were dead so I would not have to deal with this loss.” Originally, the item had four response options (0 = Not at all, 1 = Several days, 2 = More than half of the days, 3 = Almost every day); however, in the current study, the responses were dichotomized as follows: the first option was recoded as no suicidal ideation, while the remaining three were coded as presence of suicidal ideation.

### Procedure

The study was conducted during the COVID-19 pandemic between January 25 and February 26, 2021. During this period of time, Latin America reached the figure of nearly 21 million people infected and almost 700,000 deaths from COVID-19 according to figures compiled by Johns Hopkins University, with Brazil being the country with the most deaths (more than 250,000), followed by Mexico (more than 115,000), Colombia (more than 50,000) and Argentina (more than 48,000) (Coronavirus Resource Center, 2021). Thus, Latin America is the second most affected region, in number of deaths, after Europe, representing almost a quarter of the deaths reported worldwide. The data were collected simultaneously in the ten participating countries, and the collection procedure was the same in all of them. The online questionnaire was designed using Google Forms and distributed by email, as well as different social media platforms (Facebook, Instagram, WhatsApp). The questionnaire included questions related to sociodemographic data, COVID-19 pandemic grief, and suicidal ideation. Only those who reported having suffered the death of a family member or friend from COVID-19 and gave informed consent participated. This information was requested in the first section of the online form. Participants completed the online survey in approximately 10 minutes. Participation in the study was voluntary and no financial compensation was received for participation. Participants were required to answer all items in the questionnaire before submitting their responses. The study was approved by the Ethics Committee of the Universidad Privada del Norte (registry number: 20213002).

### Data Analysis

First, preliminary analyses were conducted at the item-level. Specifically, descriptive statistics and single-group confirmatory factor analyses (CFAs) were examined in each country separately. A robust maximum likelihood estimator (MLR) was used (Yuan & Bentler, 2000). The fit of a CFA was evaluated following widely used indices that complemented the chi-square (χ^2^) test: the comparative fit index (*CFI*), the Tucker-Lewis index (*TLI*), the root-mean-square error of approximation (*RMSEA*), as well as the standardized root-mean-square residual (*SRMR*). A non-significant χ^2^ test, *CFI* > .95, *TLI* > .95, *RMSEA* < .06, and *SRMR* < .08 were considered evidence of good fit (Hu & Bentler, 1999). However, it should be noted that the *RMSEA* tends to perform poorly in models with small degrees of freedom (Kenny et al., 2015). Thus, this index should be interpreted with caution in the present study. The *CFI*, *TLI*, and *RMSEA* values were computed using specialized formulae developed for situations in which a robust estimator is used (Brosseau-Liard et al., 2012; Brosseau-Liard & Savalei, 2014).

Second, multi-group CFAs were used to examine factorial invariance across country and gender (again using a MLR estimator). Sequential constraints were added to a baseline (configural) model: equal loadings (metric invariance), equal intercepts (scalar invariance), and equal latent means. A model was judged to be non-invariant if it had a significant Δχ^2^ test and a |Δ*CFI*| > .01 (Cheung & Rensvold, 2002). Given that moderate unbalance was present between some groups, a special subsampling approach with 100 replications was followed (Yoon & Lai, 2018).

Finally, the association between PGS scores and suicidal ideation was evaluated from the odds ratio (*OR*) obtained from a logistic regression. An adjusted *OR* (*aOR*) was also computed after controlling for country, age, and gender. Next, a receiver operating characteristic (*ROC*) curve was plotted, and the area under the curve (*AUC*) was quantified. Following Hosmer et al. (2013), an *AUC* ≥ .90 was considered outstanding. Finally, an optimal cutoff score of the PGS was identified by maximizing sensitivity and specificity to detect individuals with suicidal ideation.

All the analyses were computed in R 4.0.3. The following specialized packages were used: lavaan 0.6–8, semTools 0.5–3, and cutpointr 1.1.0.

## Results

### Preliminary Analyses

Table 3 presents the descriptive statistics of the PGS items across countries. All of them had large skewness and kurtosis values, as can be expected for the construct under study. Item 1 (*I wished to die in order to be with the deceased*) was the one that showed the greatest deviation from normality, as well as the lowest mean values in most countries.

**Table 3.** Item-Level Descriptive Statistics of the Pandemic Grief Scale.

Country	Statistic	Pandemic Grief Scale item				
		1	2	3	4	5
Bolivia (*n* = 156)	*M*	0.23	0.54	0.43	0.42	0.33
	*SD*	0.64	0.88	0.76	0.76	0.67
	*g_1_*	3.11	1.59	1.80	1.73	2.12
	*g_2_*	9.49	1.58	2.53	2.11	4.19
Brazil (*n* = 206)	*M*	0.17	0.41	0.40	0.28	0.34
	*SD*	0.59	0.74	0.78	0.68	0.79
	*g_1_*	3.96	1.92	2.10	2.69	2.36
	*g_2_*	15.26	3.30	3.74	6.86	4.57
Chile (*n* = 179)	*M*	0.23	0.42	0.36	0.22	0.34
	*SD*	0.69	0.78	0.75	0.61	0.73
	*g_1_*	3.07	1.91	2.18	2.88	2.17
	*g_2_*	8.55	2.92	4.05	7.75	3.92
Colombia (*n* = 215)	*M*	0.26	0.43	0.39	0.40	0.33
	*SD*	0.72	0.85	0.79	0.80	0.73
	*g_1_*	2.94	1.96	2.06	1.90	2.31
	*g_2_*	7.71	2.74	3.30	2.44	4.49
Ecuador (*n* = 295)	*M*	0.31	0.60	0.55	0.51	0.46
	*SD*	0.72	0.88	0.85	0.86	0.79
	*g_1_*	2.50	1.29	1.44	1.62	1.73
	*g_2_*	5.59	0.55	1.11	1.57	2.22
El Salvador (*n* = 437)	*M*	0.33	0.57	0.56	0.51	0.47
	*SD*	0.78	0.93	0.92	0.89	0.88
	*g_1_*	2.35	1.49	1.51	1.67	1.85
	*g_2_*	4.45	0.97	1.08	1.59	2.27
Guatemala (*n* = 171)	*M*	0.24	0.49	0.42	0.35	0.41
	*SD*	0.68	0.88	0.82	0.77	0.85
	*g_1_*	2.98	1.63	1.88	2.15	1.96
	*g_2_*	8.06	1.40	2.42	3.58	2.56
Mexico (*n* = 202)	*M*	0.31	0.65	0.53	0.46	0.43
	*SD*	0.77	0.99	0.90	0.88	0.83
	*g_1_*	2.55	1.36	1.61	1.81	2.02
	*g_2_*	5.43	0.56	1.44	1.99	3.20
Paraguay (*n* = 441)	*M*	0.28	0.39	0.39	0.38	0.35
	*SD*	0.73	0.79	0.79	0.78	0.74
	*g_1_*	2.69	2.06	2.08	2.08	2.21
	*g_2_*	6.19	3.31	3.41	3.39	4.08
Peru (*n* = 234)	*M*	0.29	0.53	0.50	0.43	0.37
	*SD*	0.68	0.86	0.86	0.83	0.77
	*g_1_*	2.51	1.58	1.69	1.97	2.18
	*g_2_*	5.71	1.55	1.88	2.87	3.95

Next, CFAs were conducted for each country separately. The unidimensional model had excellent fit in most countries, except for Colombia, Ecuador and Paraguay, where the fit was nonetheless acceptable (Table 4). Modification indices were examined for these countries, but no theoretically defensible re-specification was identified. Thus, the unidimensional model was taken as a baseline for the following invariance analyses. The standardized factor loadings, as well as the internal consistency reliability estimates, are presented in Table 5.

**Table 4.** Single-Group Confirmatory Factor Analyses of the Pandemic Grief Scale.

Country	χ²	*df*	*p*	*CFI*	*TLI*	*RMSEA*	*SRMR*
Bolivia	3.81	5	.577	1	1.02	0	.03
Brazil	8.06	5	.153	.98	.96	.08	.04
Chile	3.83	5	.574	1	1.01	0	.02
Colombia	17.51	5	.004	.96	.92	.16	.04
Ecuador	22.09	5	.001	.97	.95	.13	.02
El Salvador	10.81	5	.055	.99	.98	.08	.02
Guatemala	10.46	5	.063	.98	.96	.12	.04
Mexico	4.51	5	.478	1	1	0	.02
Paraguay	14.91	5	.011	.98	.96	.11	.03
Peru	9.55	5	.089	.99	.98	.09	.03

*Note.* The estimator was robust maximum likelihood (MLR). Robust versions of the *CFI*, the *TLI*, and the *RMSEA* are reported (Brosseau-Liard et al., 2012; Brosseau-Liard & Savalei, 2014). *CFI* = comparative fit index; *RMSEA* = root-mean-square error of approximation; *SRMR* = standardized root-mean-square residual.

**Table 5.** Factor Loadings and Internal Consistency Reliability of the Pandemic Grief Scale.

Country	Items					ɑ	ω
	1	2	3	4	5		
Bolivia	.68	.59	.77	.59	.77	.80	.80
Brazil	.71	.71	.80	.56	.66	.81	.82
Chile	.87	.78	.85	.52	.81	.88	.89
Colombia	.89	.73	.83	.65	.93	.90	.90
Ecuador	.79	.85	.91	.70	.84	.91	.91
El Salvador	.81	.88	.89	.72	.86	.92	.92
Guatemala	.80	.83	.96	.49	.88	.89	.90
Mexico	.77	.77	.89	.70	.83	.89	.90
Paraguay	.80	.86	.87	.69	.84	.91	.91
Peru	.89	.79	.83	.73	.87	.91	.91

*Note.* Completely standardized factor loadings are presented. The estimator of the confirmatory factor analysis was maximum likelihood.

### Factorial Invariance

First, invariance was examined across countries. As presented in Table 6, evidence of scalar invariance was found both under the Δχ^2^ and the Δ*CFI* criteria. When the equal means restriction was added, model fit did not worsen to a significant degree. Thus, latent means were assumed to be similar across countries. Second, invariance was also tested across genders and evidence of scalar invariance was found (Table 5). The model with equal means showed a significant reduction in fit according to the Δχ^2^ criterion, but not to the pragmatic Δ*CFI*, thus suggesting that the difference was negligible in magnitude.

**Table 6.** Measurement and Structural Invariance of the Pandemic Grief Scale.

Grouping variable	Model	χ²	*df*	*CFI*	*RMSEA*	Δχ²	Δ*df*	Δ*CFI*
Country	Configural	85.73**	50	.98	.10			
	Metric	123.14**	86	.98	.08	38.63	36	0
	Scalar	172.68**	122	.98	.07	47.91	36	0
	Equal means	185.97**	131	.98	.07	13.62	9	0
Gender	Configural	29.87***	10	.99	.08			
	Metric	32.98**	14	.99	.06	2.31	4	0
	Scalar	39.78**	18	.99	.05	4.04	4	0
	Equal means	44.39***	19	.99	.06	7.17**	1	0

*Note.* Yoon and Lai’s (2018) subsampling approach with 100 replications was followed in both cases. Robust versions of the *CFI* and the *RMSEA* are reported (Brosseau-Liard et al., 2012; Brosseau-Liard & Savalei, 2014). *CFI* = comparative fit index; *RMSEA* = root-mean-square error of approximation.

* *p* < .05. **p < .01. ***p < .001.

### Association With Suicidal Ideation and Proposed Cut-Off

In order to examine associative evidence of validity, we observed how well the PGS scores predicted suicidal ideation cross-sectionally. We found an *OR* = 1.90, 95% CI [1.80, 2.02], meaning that an increase in one point on the PGS was related to almost a twofold increase in the odds of suicidal ideation. These results were unchanged after adjusting for country, age and gender, *aOR* = 1.90, 95% CI [1.79, 2.01].

**Figure 1.**
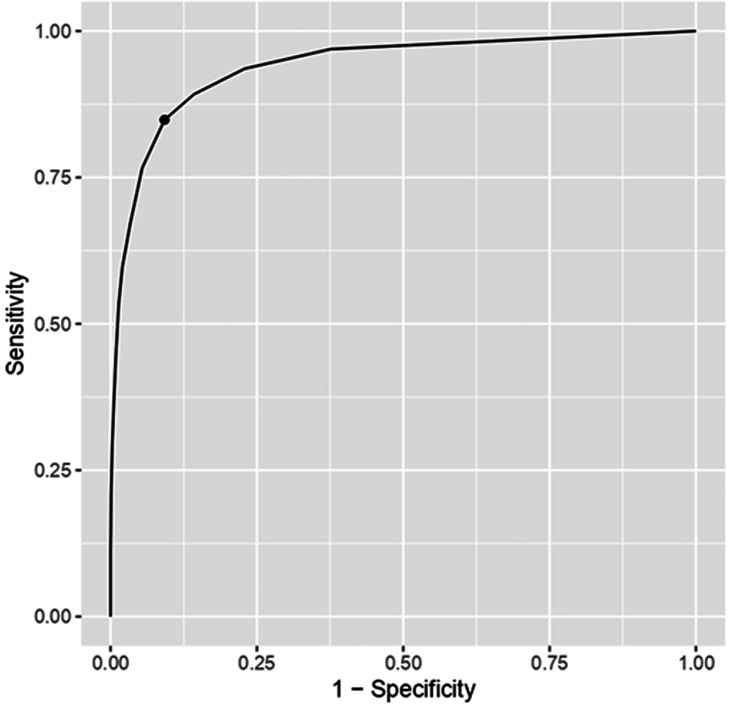
ROC Curve of the PGS Total Score as Predictor of Suicidal Ideation.

**Figure 2.**
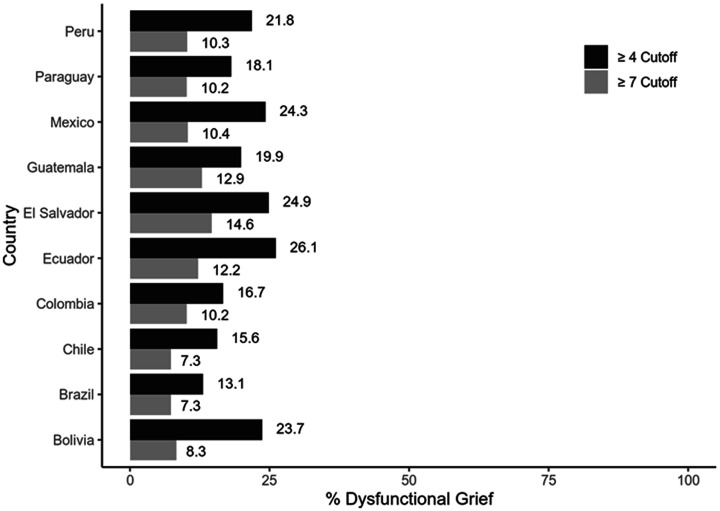
Percentage of Individuals Scoring Above Two Alternative Cutoffs of the Pandemic Grief Scale.

Given the strong association between the PGS scores and suicidal ideation, we considered the possibility that the former may be used to identify possible cases of the latter. The *ROC* curve is presented in Figure 1; the *AUC* was .94, suggesting outstanding discrimination. Different cutoff values were examined until we found one that gave an optimal trade-off between sensitivity and specificity. We propose that scores greater than or equal to 4 may be used to identify individuals presenting suicidal ideation (sensitivity = .85, specificity = .91). Figure 2 shows the percentages of participants who scored above this value (as well as the original ≥7 cutoff) in each country.

## Discussion

The aim of the present study was to examine the cross-cultural measurement invariance (MI) of the Pandemic Grief Scale (PGS; Lee & Neimeyer, 2020) and its relation to suicidal ideation in samples from 10 Latin American countries. The PGS is a simple and easy instrument to screen for the presence of dysfunctional grief related to the COVID-19 pandemic, making it a useful tool for the evaluation of mental health indicators. However, to date there are no studies evaluating the MI of the PGS in the general population in countries other than the one in which it was originally developed, such as the Latin American countries in this study. In general, the PGS demonstrated adequate psychometric properties in all countries evaluated. Specifically, the results of the CFA show that the PGS presents a unidimensional factor structure in all the countries involved. Although the RMSEA values were higher than recommended in some countries, such as Colombia, Ecuador, Guatemala, Paraguay and Peru (Kline, 2015; Schumacker & Lomax, 2015), this was to be expected, since the RMSEA tends to perform poorly in factor models with few degrees of freedom (Kenny et al., 2015; Taasoobshiraz & Wang, 2016). On the other hand, the results for the reliability of the PGS were also promising in all samples analyzed, where the values of the alpha and omega coefficients ranged between .80 and .92, which is an adequate range for internal consistency. The above confirms previous findings in the United States (Lee & Neimeyer, 2020), European countries such as Turkey (Evren et al., 2021) and Poland (Skalski et al., 2021), and a single-country study in Latin America (Caycho-Rodríguez et al., 2021a).

In addition, full configural, metric, and scalar invariance was established among the 10 countries assessed. The results with respect to configural invariance indicate that, across the 10 countries, the concept of COVID-19 pandemic-related dysfunctional grief can be assessed using all five PGS items and that these same items can best be represented in a single latent variable. Therefore, it is suggested that the concept of COVID-19 pandemic-related dysfunctional grief as assessed in this study has uniform meaning across the 10 countries included in the analysis. Similarly, the presence of metric invariance showed that all items had similar factor loadings in all samples. Thus, the items are interpreted and responded to in a similar way across countries. Finally, scalar invariance shows that the observed differences in item means reflect true differences in the latent construct and not measurement errors. These findings regarding cross-cultural MI have important implications, as they ensure that the PGS would be assessing dysfunctional grief related to the COVID-19 pandemic in a similar way across countries. This is even more relevant in cultural regions, such as Latin America, where expressions of grief and crying during grief are considered healthy and encouraging (Hardy-Bougere, 2008; Parry & Ryan, 1996). These findings are inserted in a line of research that has received much attention from researchers for some years: the scientific study of grief within different sociocultural contexts (Stroebe et al., 2001a).

Additionally, it is suggested that gender would explain part of the variability observed in grief (Lundorff et al., 2020). In this sense, the MI of the PGS was also assessed between men and women in the 10 countries evaluated, indicating that the scale would assess dysfunctional grief related to the COVID-19 pandemic in a similar way between male and female groups. This finding is consistent with the original study in the U.S. population, where the construct is reported to be measured in the same way across the gender variable (Lee & Neimeyer, 2020). This evidence that men and women in the 10 countries understand COVID-19 grief in the same way could be explained by social role theory (Eagly et al., 2000). Latin American countries share many cultural beliefs about what social roles men and women should occupy within a society (Stelzer et al., 2019). Thus, it is customary that women tend to be more expressive in their pain and trust others more than men (Stroebe et al., 2001b). This finding is important for comparative studies of gendered grief where information is contradictory. For example, part of the scientific literature points to female gender as a potential risk factor for intense and prolonged grief reactions (Burke & Neimeyer, 2012; Heeke et al., 2019; Lee & Neimeyer, 2020); while others point out that gender does not significantly moderate grief prevalence rates following a loss (Djelantik et al., 2020; Lundorff et al., 2017). Even a longitudinal study points out that men would express prolonged grief as an acute and decreasing reaction; while women present an increasing grief reaction (Lundorff et al., 2020).

Another analysis suggested that each one-point increase in PGS scores was associated with a nearly twofold increase in the odds of suicidal ideation in people across the 10 countries, even after holding country, age, and sex variables constant. This aligns with other findings indicating an increase in suicidal ideation resulting from grief reactions ( Doering & Eisma, 2016; Halford et al., 2020; Reger et al., 2020; Wand et al., 2020). This result can be explained from the interpersonal theory of suicide, where the belief of being a burden and the perception that other people would benefit from our death, together with loneliness, social isolation and poor social support, contribute to the presence of suicidal ideations (Van Orden et al., 2010). Likewise, indicating that dysfunctional grief related to COVID-19 predicts suicidal ideation would suggest that identifying suicidal ideation during grief would be of utmost importance within therapeutic interventions in each country (Jang et al., 2018).

The present study also proposed that scores greater than or equal to 4 on the PGS would allow for identifying people with suicidal ideation. Based on this, it was identified that Ecuador and El Salvador were the countries with the highest probability of people with suicidal ideation, while Brazil presented the lowest probability. The variation in suicidal ideation between countries during the COVID-19 pandemic has been reviewed previously, and it has been suggested that the differences may be related to public spending and financial resources available to citizens (Cheung et al., 2021). In this regard, lower incomes, poor social support, and deficits in medical care may increase the likelihood of suicidal ideation due to a lack of hope for the future during the COVID-19 pandemic. On the other hand, these same countries had the highest prevalence rates of dysfunctional grief related to COVID-19 along with Guatemala. Overall, the percentages of dysfunctional grief related to COVID-19 ranged from 7.3% (Brazil and Chile) to 14.6% (El Salvador), which are lower than those reported in U.S. individuals which ranged from 56.6% to 66% (Lee & Neimeyer, 2020; Lee et al., 2021). This was to be expected as the United States is so far, the country with the most deaths from COVID-19, which could have led to an unprecedented grief overload (Kokou-Kpolou et al., 2020). According to the latest reports of the Coronavirus Resource Center, the United States has more than 606,300 thousand deaths (https://coronavirus.jhu.edu/map.html). The fact that Ecuador and El Salvador have the highest prevalence of dysfunctional grief is associated with the number of deaths and the management of the pandemic at the time of the study in each country. Thus, at the time of data collection, in El Salvador, there were 63,344 confirmed cases of COVID-19, of which 1,986 were reported as deaths; while the type of transmission of the disease was classified as “Local”, specifically at the community level (phase III). In addition, the country was in phase II of economic reactivation, which led to an increase in the number of infections at the national level. In the case of Ecuador, during the same period of time, 16,780 deaths from COVID-19 were reported, which led Ecuador to face one of the highest periods of hospitalizations for this disease, characteristic of a third mortality wave of this health emergency. In Guatemala, at the time information was collected for this study, there were more than 164,746 confirmed cases and 5,989 deaths. This scenario has also led to the emergence of negative mental health outcomes in these countries in terms of anxiety, stress and a worsening of pre-existing mental disorders (Alonzo et al., 2021; Orellana, & Orellana, 2020; Terán-Pérez et al., 2021). These prevalence rates could have significant physical and mental health consequences that will necessitate preventive and supportive interventions for bereaved persons (Kokou-Kpolou et al., 2020).

Although the present study constitutes a pioneering effort in the scientific literature on the cross-cultural validity of instruments to measure mental health symptoms during the COVID-19 pandemic, the results should be evaluated in light of its limitations. First, most participating countries were from South America (Bolivia, Brazil, Chile, Colombia, Ecuador, Paraguay and Peru) and only three from Central and North America (El Salvador, Guatemala and Mexico). Therefore, future studies should include more Latin American countries, especially from Central America. In addition, due to the non-probability convenience sampling used to select participants, the national samples included in this study are not representative of each of the countries. This limits the possibility of generalizing the results to the entire population of each of the countries. Furthermore, even though the ten countries present different social and cultural backgrounds, future studies should be conducted with samples from other Western and Eastern countries to corroborate the generalizability of the PGS across different cultural contexts. Also, there are different sample sizes across countries and some imbalances in the number of participants according to certain sociodemographic variables such as gender, where significantly more men than women participated in the study. Thus, it is possible that the same levels of variability in expressions of grief across countries were not captured, which would further limit the generalizability of the findings. While it has been suggested that differences in sample size between groups could bias the results when conducting an MI analysis (Brown, 2006); the use, in this study, of the procedure suggested by Yoon and Lai (2018) has helped moderate the impact of different sample sizes. Even so, it is important for future studies to have larger samples which are equivalent in number and balanced by gender from Latin American countries to obtain firmer conclusions.

Additionally, the use of an online survey caused a selection bias in favor of people with Internet access and experience in answering this type of survey. This means that the sample is not completely generalizable to the population of people who have suffered the loss of a loved one due to COVID-19 in each of the countries involved. Another limitation of this study involves the relevance of the death wish item of the Pandemic Grief Scale (Lee & Neimeyer, 2020) for some respondents. Specifically, the death wish item, “I wished to die in order to be with the deceased,” may not be relevant for respondents who do not believe in the existence of an afterlife and interpret this statement in those terms. Furthermore, participants were not asked if they believe or do not believe in any religion. This information would be useful for a study on the impact of specific religious beliefs on grief management, as previous studies have indicated (Feldman et al., 2016). This type of information would help to better understand the low scores shown by participants for item 1 (“I wished to die in order to be with the deceased”), which presupposes that respondents believe in some kind of life after death. Future research should empirically examine this potential issue.

Although the recoding of the single item options of suicidal ideation (the first option was coded as no suicidal ideation, while the remaining three were coded as presence of suicidal ideation) may imply that some detailed information about the different levels of suicidal ideation was lost, it is also true that having two categories of suicidal ideation facilitates the interpretation of the results for decision making. Indeed, measuring suicide-related variables as binary is common practice in public health research (e.g., Kappel et al., 2021; Xu et al., 2020). Furthermore, given that the original measure had only four categories, it would have been problematic to treat it as continuous. Consequently, for both substantive and methodological reasons, it was decided to treat suicidal ideation as a dichotomous variable. Future studies should test the usefulness of this strategy. Finally, bereavement experience and suicidal ideation, as well as some sociodemographic variables were assessed by self-report measures, which could lead to the presence of social desirability bias and an underreporting of information.

Despite these limitations, the current findings are encouraging with respect to the MI of the PGS in 10 countries. The strengths of the study were its use of two language versions of the PGS (Spanish and Portuguese) including individuals from economically, culturally and religiously diverse countries. In this way, it was possible to cover a greater range of people and countries, as well as to extend the findings to multicultural contexts. In conclusion, the PGS showed good psychometric properties in samples from Bolivia, Brazil, Chile, Colombia, Ecuador, Paraguay, El Salvador, Guatemala and Mexico. In addition, it was established that the cross-cultural MI of the PGS remains strong despite the greater number of countries evaluated, which provides solid evidence of cross-cultural validity of the scale. Although further studies are needed to confirm the current psychometric findings, the PGS can be considered a reliable measure that, because of its brevity, can be used in large-scale cross-cultural studies for rapid and cost-effective assessment of pandemic-related grief. Although the associations between psychological conditions and attitudes during the COVID-19 pandemic are complex, the PGS may be useful in epidemiological studies as a screening tool to identify individuals who are at risk of experiencing suicidal thoughts. Suicide and suicidal ideation are a global concern, especially during a pandemic. Finally, it may also be useful to evaluate which interventions have had an effect on the management of pandemic-related grief and the possibility of growth (Doka, 2021). Highly stressful circumstances are known to contribute to positive and deeply meaningful shifts in the way people view the world.
